# Supplementary material for: Pan-cancer analysis of the effect of biopsy site on tumor mutational burden observations
Source: Commun Med (Lond). 2021 Dec 2;1:56. doi: 10.1038/s43856-021-00054-8 (PMC9053207; doi:10.1038/s43856-021-00054-8)
Supplement: Supplementary file 6 — Description of Additional Supplementary Files [file 43856_2021_54_MOESM6_ESM.pdf]

Description of Additional Supplementary Files

File Name: Supplementary Data 1

Description: Distribution of samples counts

File Name: Supplementary Data 2

Description: Average TMB difference between metastatic and local tissue across multiple cancer types.

File Name: Supplementary Data 3

Description: Distribution of patients being assessed as TMB-High (>10 mut/Mb) per cancer type and tissue of biopsy.

File Name: Supplementary Data 4

Description: Average TMB difference between metastatic and local tissue across multiple cancer types, controlling for metastatic state

File Name: Supplementary Data 5

Description: Distribution of sample counts for patients in the same metastatic state
