# Supplementary material for: Pan-cancer analysis of the effect of biopsy site on tumor mutational burden observations
Source: Commun Med (Lond). 2021 Dec 2;1:56. doi: 10.1038/s43856-021-00054-8 (PMC9053207; doi:10.1038/s43856-021-00054-8)
Supplement: Supplementary file 8 — Reporting Summary [file 43856_2021_54_MOESM8_ESM.pdf]

nature research

Corresponding author(s): DBPR COMMSMED-21-0109B

Last updated by author(s): Nov 2, 2021

Reporting Summary

Nature Research wishes to improve the reproducibility of the work that we publish. This form provides structure for consistency and transparency in reporting. For further information on Nature Research policies, see our Editorial Policies and the Editorial Policy Checklist.

Statistics

For all statistical analyses, confirm that the following items are present in the figure legend, table legend, main text, or Methods section.

n/a	Confirmed	
☐	☒	The exact sample size (n) for each experimental group/condition, given as a discrete number and unit of measurement
☐	☒	A statement on whether measurements were taken from distinct samples or whether the same sample was measured repeatedly
☐	☒	The statistical test(s) used AND whether they are one- or two-sided<br>*Only common tests should be described solely by name; describe more complex techniques in the Methods section.*
☐	☒	A description of all covariates tested
☐	☒	A description of any assumptions or corrections, such as tests of normality and adjustment for multiple comparisons
☐	☒	A full description of the statistical parameters including central tendency (e.g. means) or other basic estimates (e.g. regression coefficient) AND variation (e.g. standard deviation) or associated estimates of uncertainty (e.g. confidence intervals)
☐	☒	For null hypothesis testing, the test statistic (e.g. F, t, r) with confidence intervals, effect sizes, degrees of freedom and P value noted<br>*Give P values as exact values whenever suitable.*
☒	☐	For Bayesian analysis, information on the choice of priors and Markov chain Monte Carlo settings
☒	☐	For hierarchical and complex designs, identification of the appropriate level for tests and full reporting of outcomes
☐	☒	Estimates of effect sizes (e.g. Cohen's d, Pearson's r), indicating how they were calculated

Our web collection on statistics for biologists contains articles on many of the points above.

Software and code

Policy information about availability of computer code

Data collection	Data points (samples) were filtered as described in Methods using custom R scripts.
Data analysis	Linear models were built using the R package emmeans. Odds Ratio were computed using R package epitools. Figures were plotted using custom R scripts.

For manuscripts utilizing custom algorithms or software that are central to the research but not yet described in published literature, software must be made available to editors and reviewers. We strongly encourage code deposition in a community repository (e.g. GitHub). See the Nature Research guidelines for submitting code & software for further information.

Data

Policy information about availability of data

All manuscripts must include a data availability statement. This statement should provide the following information, where applicable:

- Accession codes, unique identifiers, or web links for publicly available datasets
- A list of figures that have associated raw data
- A description of any restrictions on data availability

Source data for the main figures in the manuscript is available as Supplementary Data 1-5. The data that support the findings of this study are available from Foundation Medicine, but restrictions apply to the availability of these data, which were used under license for the current study, and so are not publicly available. Academic researchers can gain access to Foundation Medicine data in this study by contacting the Foundation Medicine authors and filling out a study review committee form. Any interested readers and their institutions' will be required to sign a data transfer agreement.

Field-specific reporting

Please select the one below that is the best fit for your research. If you are not sure, read the appropriate sections before making your selection.

☒ Life sciences ☐ Behavioural & social sciences ☐ Ecological, evolutionary & environmental sciences

For a reference copy of the document with all sections, see nature.com/documents/nr-reporting-summary-flat.pdf

Life sciences study design

All studies must disclose on these points even when the disclosure is negative.

Sample size	The cohort is retrospective observational and thus the sample size is simply the tally of all available samples.
Data exclusions	Each specimen was hand reviewed by a pathologist to ensure it was suitable for sequencing and we kept only specimens with >=30% tumor purity. We also ignored specimens from tissues that had fewer than 50 specimens in total.
Replication	The entirety of the analysis was double-coded by a third party that did not have access to the original analysis
Randomization	Randomization was not applicable
Blinding	The cohort is retrospective observational and thus blinding is not applicable

Reporting for specific materials, systems and methods

We require information from authors about some types of materials, experimental systems and methods used in many studies. Here, indicate whether each material, system or method listed is relevant to your study. If you are not sure if a list item applies to your research, read the appropriate section before selecting a response.

Materials & experimental systems

n/a	Involved in the study	
☒	☐	Antibodies
☒	☐	Eukaryotic cell lines
☒	☐	Palaeontology and archaeology
☒	☐	Animals and other organisms
☒	☐	Human research participants
☐	☒	Clinical data
☒	☐	Dual use research of concern

Methods

n/a	Involved in the study	
☒	☐	ChIP-seq
☒	☐	Flow cytometry
☒	☐	MRI-based neuroimaging

Clinical data

Policy information about clinical studies

All manuscripts should comply with the ICMJE guidelines for publication of clinical research and a completed CONSORT checklist must be included with all submissions.

Clinical trial registration	Not a clinical trial
Study protocol	Not a clinical trial
Data collection	Multi-center, real-world genetic testing in clinical practice
Outcomes	Outcomes not addressed in this paper
